# Mosquito mutations F290V and F331W expressed in acetylcholinesterase of the sand fly *Phlebotomus papatasi* (Scopoli): biochemical properties and inhibitor sensitivity

**DOI:** 10.1186/s13071-025-06691-5

**Published:** 2025-02-18

**Authors:** Kevin B. Temeyer, Fan Tong, Kristie G. Schlechte, Qiao-hong Chen, Paul R. Carlier, Adalberto Á. Pérez de León, Jeffrey R. Bloomquist

**Affiliations:** 1 Knipling-Bushland US Livestock Insects Research Laboratory, Agricultural Research Service, US Department of Agriculture, 2700 Fredericksburg Road, Kerrville, TX 78028-9184 USA; 2 Department of Entomology and Nematology, Emerging Pathogens Institute, University of Florida, 2055 Mowry Road, PO Box 100009, Gainesville, FL 32610-00009 USA; 3 Department of Chemistry, Virginia Tech, 900 West Campus Drive, 480 Davidson Hall, Blacksburg, VA 24061-0001 USA; 4 Department of Pharmaceutical Sciences, University of Illinois Chicago, 833 S Wood St, Chicago, IL 60612 USA; 5 San Joaquin Valley Agricultural Sciences Center, US Department of Agriculture—Agricultural Research Service, 9611 S. Riverbend Ave., Parlier, CA 93648 USA

**Keywords:** Anticholinesterase inhibition, Carbamate, Tacrine dimer, Mosquito mutations, G119S, F290V, F331W

## Abstract

**Background:**

The Old World sand fly, *Phlebotomus papatasi* (Scopoli), a vector of zoonotic cutaneous leishmaniasis, is usually controlled by insecticides, including anticholinesterases. Previous studies have revealed 85% amino acid sequence identity of recombinant *P. papatasi* acetylcholinesterase (r*Pp*AChE1) to mosquito AChE. They identified synthetic carbamates that selectively inhibited r*Pp*AChE1 and circumvented the G119S mutation responsible for high-level resistance to anticholinesterases. This study reports the construction, baculovirus expression, and biochemical properties of r*Pp*AChE1 containing the F290V and F331W orthologous mutations from mosquitoes.

**Methods:**

Recombinant *Pp*AChE1 enzymes with or without the F290V, F331W, and G119S orthologous mosquito mutations were expressed in *Sf*21cells utilizing the baculoviral system. Ellman assays determined changes in catalytic properties and inhibitor sensitivity resulting from wild type and mutant r*Pp*AChE1 containing single or combinations of orthologous mosquito mutations.

**Results:**

Each of the orthologous mutations (F290V, F331W, and G119S) from mosquito AChE significantly reduced inhibition sensitivity to organophosphate or carbamate pesticides, and catalytic activity was lost when they were expressed in combination. Novel synthetic carbamates were identified that significantly inhibited the r*Pp*AChEs expressing each of the single orthologous mosquito mutations.

**Conclusions:**

These novel carbamates could be developed as efficacious insecticides, with improved specificity and safety for use in sand fly or mosquito populations expressing the mutant AChEs.

**Graphical Abstract:**

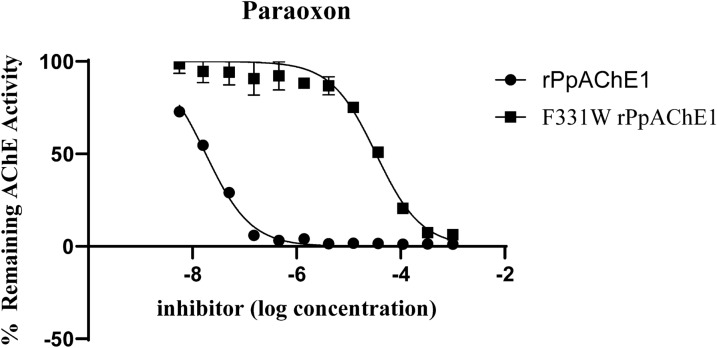

## Background

Approximately one million people are affected annually by cutaneous leishmaniasis, a widespread but neglected disease of intertropical and temperate regions [1–3]. Leishmaniasis results from *Leishmania* protozoans that are spread by the blood feeding of several sand fly species, including *Phlebotomus papatasi* (Scopoli), a primary vector of zoonotic cutaneous leishmaniasis in the Middle East, Asia, Africa, and Southern Europe [4–8]. In addition, sand fly bites significantly impacted US military operations and readiness in Iraq and Afghanistan [9–12]. Control technologies to reduce vector transmission of leishmaniasis include reducing rodent habitat or treatment of host rodents with insect growth regulators to reduce sand fly populations, use of attractive toxic sugar baits, domiciliary insecticide sprays, and insecticide-treated bed nets to decrease the number of sand fly bites to humans [4, 13–21].

Evidence for organophosphate and carbamate resistance was reported in sand flies [13, 16, 22, 23]. Point mutations in AChEs of insect disease vectors cause reduced sensitivity to anticholinesterases [24–26]. The primary mutation responsible for very high-level anticholinesterase resistance in *Anopheles gambiae* and *Culex pipiens* AChE was identified as G119S, using the original numbering system from *Torpedo californica* [27–29]. Our subsequent experiments demonstrated that the orthologous G119S mutation engineered into recombinant *P. papatasi* AChE1 (r*Pp*AChE1) caused high levels of resistance (452- to 19,213-fold) to commercial anticholinesterase insecticides, but much less (18- to 64-fold) to small core pyrazole carbamates [30].

Pyrethroids, the cholinesterase-inhibiting organophosphates, and carbamates are among the insecticides approved by the World Health Organization Pesticide Evaluation Scheme (WHOPES) for *P.* *papatasi* chemical control [31]. We previously reported that recombinant acetylcholinesterase (rAChE) of *P. papatasi* (r*Pp*AChE1) has 85% amino acid sequence identity to *Culex pipiens* and *Aedes aegypti* mosquito AChEs [32]. Our further characterization of r*Pp*AChE1 involved responses to carbamate and organophosphate catalytic site-directed inhibitors, as well as known (e.g., donepezil) and novel bivalent inhibitors occupying both the catalytic and peripheral sites [33]. These studies demonstrated that carbamate insecticides previously found to be selective inhibitors of *Anopheles gambiae* mosquito AChE were in some cases also 100- to 600-fold selective for r*Pp*AChE1 compared with human or bovine AChE [33].

Here, we report the construction and baculoviral expression of recombinant *Pp*AChE1 containing F290V and F331W mutations, which were previously demonstrated to underlie target site resistance to cholinesterase inhibitors in mosquitoes [34–36]. In addition, we report experiments that characterized biochemical properties and inhibitor sensitivities of the r*Pp*AChE1, including probing the gorge geometry of all three mutants (G119S, F290V, and F331W) with a series of tacrine dimers, as was previously reported for the wild type r*Pp*AChE [33]. We demonstrated that several of the novel synthetic carbamates were efficacious inhibitors of rPpAChE1 constructs containing the G119S, F290V, and F331W mutations responsible for resistance to pesticides targeting mosquito cholinesterases. The novel synthetic carbamates were previously shown to exhibit significantly improved specificity for arthropod cholinesterases and improved mammalian safety. Results reported here provide further evidence that in addition to *P. papatasi* and mosquitoes, the novel synthetic carbamates with improved specificity and safety could be developed to control other important arthropod disease vectors resistant to cholinesterase-inhibiting pesticides.

## Methods

### Sand flies, RNA, cDNA synthesis, agarose gel electrophoresis, and r*Pp*AChE1 expression

Sand flies used in this study were obtained from a laboratory *P. papatasi* colony maintained at the USDA-ARS, Knipling-Bushland US Livestock Insects Research Laboratory in Kerrville, TX, USA. Sand fly colony maintenance, RNA preparation, synthesis of cDNA, agarose gel electrophoresis, and expression of recombinant *Pp*AChE1 were all performed as previously described [30].

### Targeted mutagenesis and baculoviral expression of r*Pp*AChE1 mutants

A baculovirus expression vector including the cDNA encoding *Pp*AChE1 [GenBank: AFP20868.1; 30] was the template used for targeted mutagenesis. A serine codon (AGC) replaced the glycine codon (GGA) at nucleotide positions 837–839 [GenBank: JQ922267] to produce the G119S orthologous mutation (*Torpedo* AChE nomenclature) in *PpAChE1* cDNA (G256S) as previously described [30].

A valine codon (GTT) replaced the phenylalanine codon (TTT) at nucleotide positions 444–446 [GenBank: JQ922267] to construct the F290V orthologous mutation (*Torpedo* AChE nomenclature) in *PpAChE1* cDNA (F425V) [32]. High-fidelity polymerase chain reaction (PCR) used Phusion HotStart DNA polymerase (New England BioLabs, Ipswich, MA, USA), primers *Pp*AChE1F290V-1341U27-Kpn1 (5′-TCCGGTACCTTTTGTACCAGTTGTAGA-3′) and *Pp*AChE1F290V-1326L27-Kpn1 (5′-AGCGGTACCTCACATATTCCGAGATTG-3′), and a template DNA (*Pp*AChE1 coding sequence cloned in pcR4 [Life Technologies, Carlsbad, CA, USA]). They were preincubated for 30 s at 98 °C followed by 25 cycles, each consisting of 10 s at 98 °C, 45 s at 65 °C, and 5 min at 72 °C, with incubation at 72 °C for 10 min. The product amplicon was digested with Kpn1 and ligated together with Kpn1-digested *Pp*AChE1 in pBlueBac4.5/V5-His baculoviral expression vector [30] using a Quick Ligation^™^ Kit (New England BioLabs) according to the manufacturer’s instructions. It was transformed into chemically competent TOP10 *E. coli* cells (Life Technologies) and plated onto L-agar plates containing 100 µg/ml carbenicillin (Sigma Chemical Co, St. Louis, MO, USA). Plasmid DNA was sequenced from transformant colonies to verify correct construction of the *Pp*AChE1 containing the F290V orthologous mutation, co-transfected into *Sf*21 insect cells with Bac-N-Blue DNA for baculovirus expression, and characterized biochemically in microplates using a modified Ellman’s assay, as previously described [30].

A tryptophan codon (TGG) replaced the phenylalanine codon (TTC) at nucleotide positions 1447–1449 [GenBank: JQ922267] to produce the F331W orthologous mutation (*Torpedo* AChE nomenclature) in *PpAChE1* cDNA (F466W) [23]. High-fidelity PCR utilized phosphorylated primers (SigmaGenosys, St. Louis, MO, USA) Pp-F331W-1436L20P (5′Phos-CTCAGTATTACTTCCCGTGA-3′) and Pp-F331W-1456U28P (5′Phos-GAGGGATACTACTGGATCATATACTATC-3′) with Phusion HotStart DNA polymerase (New England BioLabs) and DNA template (*Pp*AChE1 in pBlueBac4.5/V5-His, [32]) preincubated for 30 s at 98 °C. This was followed by 25 cycles, each consisting of 10 s at 98 °C, 45 s at 65 °C, and 5 min at 72 °C, with a final 10 min incubation at 72 °C. The product amplicon was ligated using a Quick Ligation™ Kit (New England BioLabs) according to the manufacturer’s instructions, transformed into chemically competent TOP10 *E. coli* cells (Life Technologies, Carlsbad, CA, USA), and plated on L-agar plates containing 100 µg/ml carbenicillin (Sigma Chemical Co, St. Louis, MO, USA). Plasmid DNA from transformant colonies was sequenced to verify the presence of the F331W orthologous mutation in the *Pp*AChE1, co-transfected with Bac-N-Blue DNA into Sf21 insect cells for baculovirus expression of the r*Pp*AChE1-F331W, and biochemically characterized in microplates using a modified Ellman’s assay as described previously [30].

Recombinant *Pp*AChE1 constructs containing multiple mutations were constructed by sequential addition of the G119S, F290V, or F331W mutations to r*Pp*AChE1 constructs as described above.

### Properties of the mosquito mutations F290V, F331W, and G119S expressed in r*Pp*AChE1

Basic biochemical properties, including the concentration of substrate producing half-maximal reaction velocity (*K*_*m*_*)* and concentrations of various inhibitors producing 50% inhibition of enzyme (IC_50_) F290V of the mosquito mutations F290V, F331W, and G119S expressed in r*Pp*AChE1, were determined as described in detail below or as previously described [30, 32].

### Anticholinesterases as probes of enzyme function

**Fig. 1:**
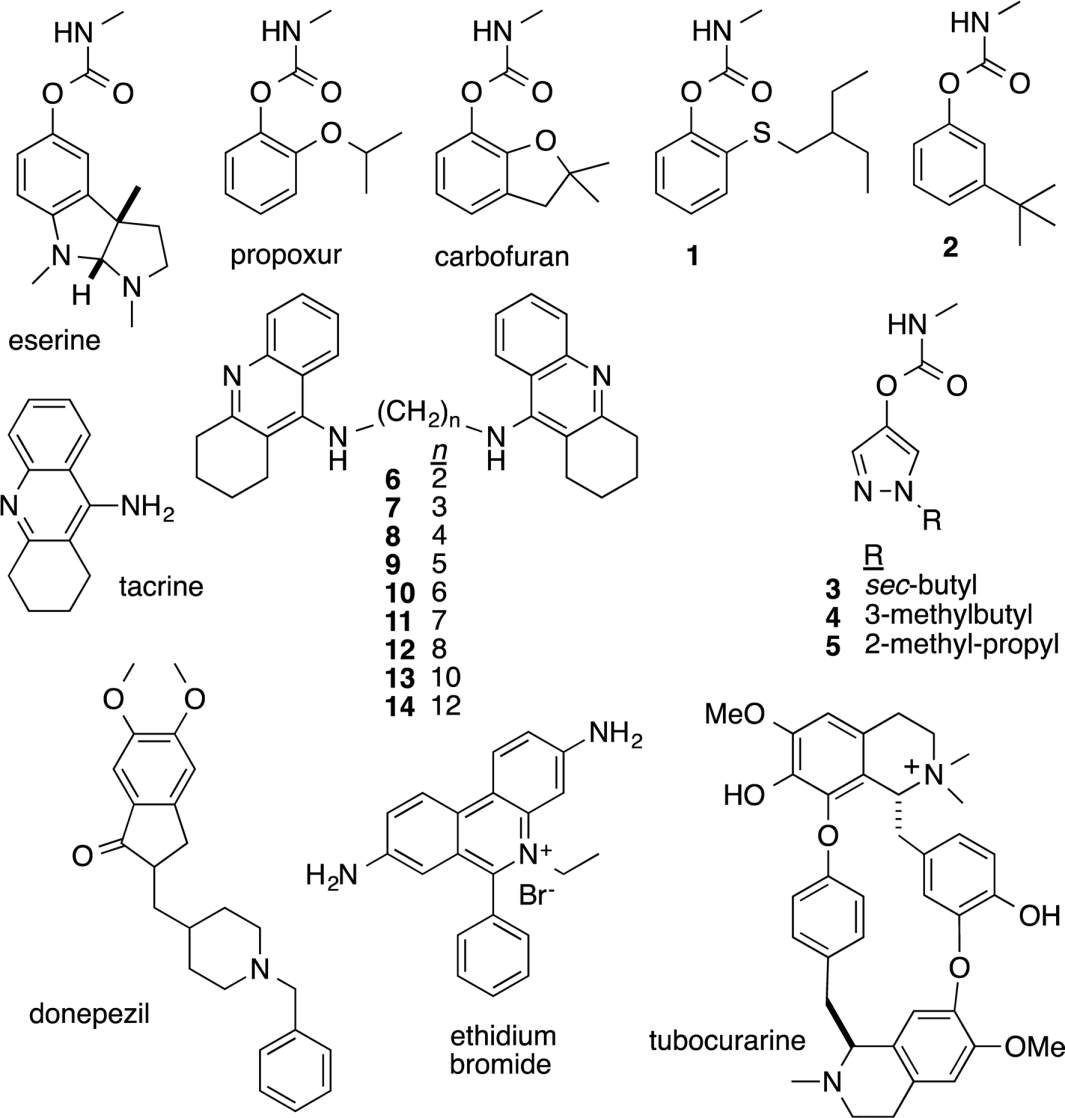
Chemical structures and names of experimental anticholinesterases mentioned in the text, with bold numbers used to designate materials without approved common names. For *bis*(*n*)-tacrines, *n* refers to the number of methylene groups in the alkyl linker

The anticholinesterases used for enzyme characterization in this study are shown in Fig. 1. Experimental pyrazole carbamates and *bis*(*n*)-tacrines were synthesized and purified using established methods [37–40] producing purities of at least 95%. Eserine (99% pure), propoxur (99%), carbofuran (99%), donepezil (98%), tacrine (99%), and ethidium bromide (95%) were purchased from Sigma-Aldrich (St. Louis, MO, USA). The peripheral site inhibitor, tubocurarine (99%), was purchased from Alfa Aesar (Ward Hill, MA, USA).

### Biochemical characterization and inhibition assays

For this study, three types of AChE inhibitors were selected to elucidate the pharmacological profiles of wild type, F290V, and F331W r*Pp*AChEs. The anticholinesterases included catalytic site inhibitors (organophosphates, carbamates, tacrine, and eserine), peripheral site inhibitors (tubocurarine and ethidium bromide), and the bivalent inhibitors *bis*(8)-tacrine, *bis*(12)-tacrine, and donepezil. Tacrine differs from the other catalytic site inhibitors because it is reversible and does not covalently bind the catalytic serine. Tacrine is bound in the choline-binding site and does not protrude into the oxyanion hole or acyl pocket [41]. The anticholinesterase compounds were dissolved in dimethyl sulfoxide (DMSO-dimethyl sulfoxide) to make stock solutions, and all enzyme assays were run with constant 0.1% DMSO as a carrier. Inhibition of r*Pp*AChE was determined using a modified Ellman assay in a 96-well plate configuration [42]. The r*Pp*AChE expression supernatants were preincubated with at least six concentrations of inhibitors for 30 min at room temperature prior to the initiation of the assay by addition of 300 µM of 5,5′-dithiobis-(2-nitrobenzoic acid) (DTNB) and 400 µM of acetylthiocholine enzyme substrate (AcSCh), each dissolved in 0.1 M of sodium phosphate buffer, pH 7.0. The kinetic reading of absorbance at 405 nm was begun immediately after the addition of DTNB and AcSCh in a Dynex Triad multimode plate reader (Dynex Technologies, Chantilly, VA, USA). Inhibitor concentration–response curves and inhibition parameters were constructed by nonlinear regression to a four-parameter logistic equation using GraphPad Prism 4.0c software (GraphPad Software, San Diego, CA, USA).

## Results

### Targeted mutagenesis and baculoviral expression of r*Pp*AChE1 mutants

Correct construction of the *rPp*AChE1s produced by targeted mutagenesis (single mutants: r*Pp*AChE1-G119S, r*Pp*AChE1-F290V, and r*Pp*AChE1-F331W; multiple combined mutants: r*Pp*AChE1-G119S-F290V, r*Pp*AChE1-G119S-F331W, r*Pp*AChE1-F290V-F331W, and f*Pp*AChE1-G119S-F290V-F331W) were verified by complete sequencing to contain the G119S, F290V, and F331W orthologous codons at the expected nucleotide positions in r*Pp*AChE1 cDNA.

**Fig. 2:**
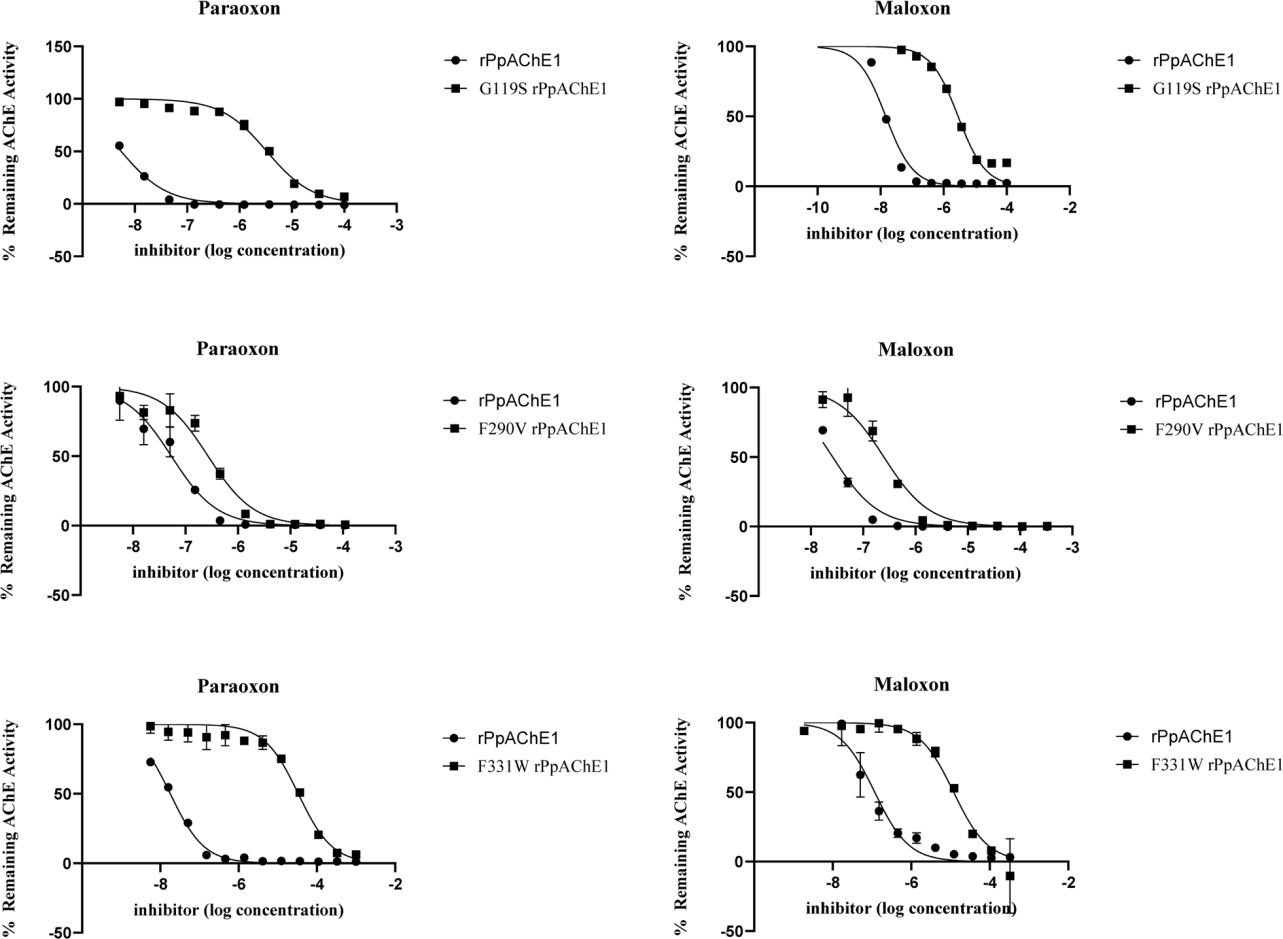
Sensitivity of the altered r*Pp*AChE1 enzymes to inhibition by paraoxon or maloxon resulting from single amino acid substitutions

Recombinant *Pp*AChE1 containing the single organophosphate-resistant (OPR) mutations G119S, F290V, or F331W each exhibited increased Michaelis–Menten values (*K*_m_) for the substrate acetylthiocholine (Table 1) and decreased sensitivity to paraoxon and maloxon inhibition (Fig. 2, Table 2). Each of the r*Pp*AChE1 constructs expressing more than one of the G119S, F290V, and F331W mutations lacked detectable AChE activity.

**Table 1:** Kinetics of AcSCh hydrolysis of wild type and mutant r*Pp*AChEs

	WT	G119S	F290V	F331W
*K*_m_ ± SEM (*n* = 3), µM	123 ± 25a	156 ± 16a	367 ± 25b	474 ± 68b
*V*max ± SEM (*n* = 3), µmol/min/mg	74 ± 6.7a	7.7 ± 2.7c	35 ± 1.2b	16 ± 2.3c
Relative *V*_max_*/K*_m_	1.0	0.082	0.158	0.056

Letters next to each kinetic value indicate results of ANOVA with Tukey’s means separation test, where values labeled by different letters are significantly different (*P* < 0.05 or less). *SEM* standard error of the mean

**Table 2:** Paraoxon and maloxon inhibition of r*Pp*AChE1 constructs expressing the G119S, F290V, and F331W mutations

r*Pp*AChE1 mutation	Paraoxon		Maloxon	
	^a^IC_50_, M (95% CI)	^b^RR	IC_50_, M (95% CI)	RR
G119S	3.82 × 10^–6^ (3.20–4.55 × 10^−6^)	1310	1.98 × 10^−6^ (1.67–2.34 × 10^−6^)	455
F290V	3.17 × 10^−7^ 2.31–4.35 × 10^−7^)	5.6	7.0 × 10^−8^ (5.09–9.76 × 10^−8^)	31
F331W	4.10 × 10^−5^ (3.22–5.24 × 10^−5^)	2035	1.80 × 10^−5^ (1.29–2.49 × 10^−5^)	344

^a^IC_50_ = inhibitor concentration (M, molar) producing 50% inhibition of enzyme activity, where (95% CI) = 95% confidence interval

^b^Resistance ratio (RR) = IC_50_ wild type/IC_50_ mutant (from Table 3)

As listed in Table 1, the *K*_m_ values for the wild type r*Pp*AChE1 and G119S, F290V, and F331W mutants were 123, 156, 367, and 474 μM AcSCh, respectively, reflecting impaired binding of substrate in the mutants. The maximal reaction velocity (*V*_max_) was also reduced in each of the expressed mutants. Considering relative *V*_max_/*K*_m_ values as a surrogate for relative *k*_cat_/*K*_m_, the G119S, F290V, and F331W mutations reduce catalytic efficiency dramatically, to between 6 and 16% of WT (Table 1). Expression of the individual mutations G119S, F290V, and F331W in r*Pp*AChE1 also reduced sensitivity to paraoxon inhibition by 1330-fold, 5.6-fold, and 2030-fold, respectively (Table 2). None of the r*Pp*AChE1 variants expressing multiple OPR mutations (G119S, F290V, and F331W) exhibited detectable AChE catalytic activity.

### Biochemical characterization and inhibition assays

As shown in Fig. 2, paraoxon and maloxon were potent inhibitors of wild type enzyme (r*Pp*AChE1), but not r*Pp*AChE1-G119S or r*Pp*AChE1-F331W. As further shown in Fig. 2 and Table 2, the r*Pp*AChE1-F290V construct was resistant to both paraoxon and maloxon, but much less so compared with the G119S or F331W mutant constructs, exhibiting resistance ratios relative to the wild type enzyme of 5.6 and 31 (F290V) compared with 1310 and 455 (G119S) and 2035 and 344 (F331W) for paraoxon and maloxon, respectively. The other anticholinesterases (Fig. 1) exhibited a wide range of potencies and resistance ratios for inhibition of the expressed mutant constructs of r*Pp*AChE1 (Tables 3, 4, 5). Calculated IC_50_ values and 95% confidence limits had correlation coefficients, *R*^2^, of at least 0.95, except for the curves with very wide confidence limits resulting from the high resistance of the G119S r*Pp*AChE to OPs and carbamates. All of the catalytic site inhibitors and bivalent inhibitors showed moderate to high potencies to inhibit wild type r*Pp*AChE enzyme activity, with IC_50_ values ranging from mid-nanomolar (e.g., propoxur and paraoxon) to sub-nanomolar concentrations (compound **7**). However, the IC_50_s for most of the compounds fell in the range of 3–76 nM (Table 3). The two peripheral site inhibitors, by comparison, had relatively low potencies for r*Pp*AChE inhibition of 17 µM (ethidium bromide) and 143 µM (tubocurarine), similar to the low affinity of propidium reported for mammalian AChE [43]. In contrast, strong resistance to the organophosphates (paraoxon and maloxon), carbofuran (*N*-methyl carbamate), and all phenyl-substituted methylcarbamates (propoxur and compounds **1**, **2**) was shown by the r*Pp*AChE-G119S, with resistance ratios over 450. Of interest, a group of alkyl-substituted pyrazole carbamates (compounds **3**–**5**), which include a smaller ring than phenyl methylcarbamates, exhibited much lower resistance ratios (18–64-fold) compared with phenyl methylcarbamates (Table 3). The other peripheral site inhibitors, bivalent inhibitors, and a catalytic site inhibitor, tacrine, exhibited the lowest resistance ratios, which were lower than **7**. Eserine was an exception, which, despite having a large pyrroloindole ring system, exhibited much less cross-resistance than the phenylcarbamates, but more than compounds showing the least cross resistance (Table 3). The current data produced with wild type r*Pp*AChE showed correlation to that previously published [24], which was different in that it used a shorter 10 min preincubation with inhibitor. Data sets collected in both studies for r*Pp*AChE were highly correlated (*R*^2^ = 0.897, *F* = 78, and *P* < 0.0001), whereas there was no correlation between r*Pp*AChE1and r*Pp*AChE1-G119S; data are shown in Table 3 (*R*^2^ = 0.151, *F* = 1.6, and *P* = 0.238).

**Table 3:** Inhibition of r*Pp*AChE1 and r*Pp*AChE1-G119S by different classes of AChE inhibitors

Compound	Inhibitor class	Wild type r*Pp*AChE1 ^a^IC_50_ (95% CI)	G119S r*Pp*AChE1 ^a^IC_50_ (95% CI)	^c^RR
Paraoxon	Acylation site	290	380,000	1310
Maloxon	Acylation site	44	20,000	455
Eserine	Acylation site	3.2 (2.6–4.0)	86 (73–102)	27
Propoxur	Acylation site	220 (147–329)	4227,000 (–)^b^	19,213
Carbofuran	Acylation site	24 (17–33)	124,000 (–)	5167
**1**	Acylation site	14 (10–19)	236,800 (7404–7575,000)	16,914
**2**	Acylation site	36 (28–48)	123,100 (–)	3419
**3**	Acylation site	13 (9.4–19)	235 (164–336)	18
**4**	Acylation site	75 (36–152)	4775 (3,048–7482)	64
**5**	Acylation site	76 (50–117)	2128 (1,267–3573)	28
Tacrine	Choline binding site	67 (56–81)	388 (318–473)	5.8
**6**	Bivalent	0.42 (0.35–0.52)	2.7 (2.3–3.2)	6.4
**7**	Bivalent	14 (13–15)	35 (28–42)	2.5
Donepezil	Bivalent	52 (39–70)	262 (202–341)	5
Tubocurarine	Peripheral site	143,200 (94,630–216,700)	661,800 (290,000–1511,000)	4.6
Ethidium bromide	Peripheral site	17,100 (13,890–21,060)	6433 (4380–9448)	0.4

^a^IC_50_ = inhibitor concentration producing 50% inhibition of activity (in nM), where (95% CI) = 95% confidence interval

^b^(–) denotes wide confidence limits from incomplete inhibition of r*Pp*AChE1-G119S

^c^Resistance ratio (RR) = IC_50_ wild type / IC_50_ mutant

**Table 4:** Inhibition of r*Pp*AChE F290V and F331W mutants by different classes of AChE inhibitors

Compound	IC_50_ (F331W), nM (95% CI)	RR F331W	IC_50_ (OPR), nM F290V (95% CI)	^a^RR F290V	RR G119S	
Acylation site inhibitors						
Eserine	85 (68–105)	17	12 (9–16)	9.2		27
Propoxur	1874 (2399–2510)	13	17,420 (13,020–23,310)	405		19,213
Carbofuran	644 (450–921)	29	2897 (2347–3576)	93		5167
** 1**	8678 (668–11,280)	271	2447 (1750–3423)	129		16,914
** 2**	845 (651–1,095)	42	1884 (1584–2240)	331		3419
** 3**	392 (327–470)	25	195 (148–257)	31		18
** 4**	1679 (1394–2022)	44	434 (329–572)	3.5		64
** 5**	6438 (5206–7961)	29	1,286 (1029–1607)	16		28
Peripheral site inhibitors						
Tubocurarine	160,800 (99,730–259,200)	6.7	26,040 (21,770–31,140)	1		4.6
Ethidium bromide	14,200 (10,220–19,730)	1.2	2969 (2419–3643)	0.2		0.4
Bivalent inhibitor						
Donepezil	253 (181–354)	4	552 (404–753)	9.7		5

Matched wild type measurements for each mutant were performed

^a^Resistance ratio (RR) = IC_50_ wild type / IC_50_ mutant

**Table 5:** Interrogation of gorge geometry by responses to tacrine and tacrine dimers of varying tether lengths

	WT	G119S	F290V	F331W
Tacrine	585 (488–701)	3872 {6.6} (3267–4646)	1,911 {3.3} (1379–2649)	2049 {3.5} (1463–2870)
*bis*(2)-tacrine	226 (192–267)	2138 {9.5} (1594–2867)	172 {0.76} (141–210)	8,842 {39} (6664–11,730)
*bis*(3)-tacrine	29 (25–33)	45 {1.6} (34–59)	13 {0.45} (11–16)	444 {15} (323–609)
*bis*(4)-tacrine	52 (44–60)	96 {1.8} (64–154)	25 {0.48} (21–30)	618 {12} (575–663)
*bis*(5)-tacrine	13 (12–15)	55 {4.2} (46–65)	2.3 {0.18} (1.7–3.2)	324 {25} (250–418)
*bis*(6)-tacrine	9.2 (7.5–11)	12 {1.3} (8.8–17)	2.7 {0.29} (2.2–3.4)	102 {11} (85–121)
*bis*(7)-tacrine	4 (3–5.3)	5.3 {1.3} (4.6–6)	2.9 {0.73} (2.2–3.8)	37 {9.3} (31–44)
*bis*(8)-tacrine	0.6 (0.5–0.7)	2.7 {4.5} (2.3–3.2)	0.3 {0.50} (0.2–0.4)	11 {18} (9.7–13)
*bis*(9)-tacrine	4.8 (3.2–7.2)	3.4 {0.7} (2.9–3.8)	1.8 {0.38} (1.5–2.1)	24 {5} (20–29)
*bis*(12)-tacrine	9.3 (8.2–10)	35 {3.8} (28–42)	6.6 {0.71} (5.5–8)	121 {13} (105–139)

Table entries are IC_50_ values in nM with (95% confidence limits). Numbers in braces {} are resistance ratios (IC_50_ mutant / IC_50_ wild type)

The r*Pp*AChE1-F290V construct exhibited significant resistance to paraoxon and maloxon, but much less than the G119S or F331W constructs (Table 2). In addition, as shown in Table 4, the F290V construct exhibits much lower sensitivity (higher RR, resistance ratios) to several of the acylation site inhibitors (propoxur, carbofuran, and compounds **1** and **2**) compared with the G119S construct, as also seen for the F331W construct. Interestingly, the F290V construct exhibited a resistance ratio of 1, indicating no difference in sensitivity to the peripheral site inhibitor, tubocurarine, compared with the wild type r*Pp*AChE1. This peripheral site inhibitor result for the F290V construct contrasts with the increased sensitivity to ethidium bromide (RR 0.2), similar to the G119S (RR0.4) construct, but different from the F331W (RR1.2) construct (Table 4).

Similarly to the G119S, the r*Pp*AChE-F331W construct showed strong resistance to the organophosphates (paraoxon and maloxon), but much lower sensitivity to carbofuran (*N*-methylphenyl carbamate), and all the synthetic carbamates. They exhibited resistance ratios generally less than 45, with the exception of compound **1**, which exhibited a resistance ratio of 271 (Table 4). All other inhibitors exhibited resistance ratios lower than 44.

Interrogation of gorge geometry by tacrine and tacrine dimers of varying length (Table 5) revealed differences between the unaltered r*Pp*AChE1 and the constructs expressing the mosquito mutations G119S, F290V, and F331W. The unaltered r*Pp*AChE1 clearly showed the greatest relative sensitivity to *bis*(8)-tacrine, as did the altered constructs expressing the G119S, F290V, and F331W mutations; however, the F290V construct exhibited increased resistance to tacrine, but increased sensitivity to all of the *bis*(*n*)-tacrine dimers. In contrast, the G119S and F331W constructs exhibited increased resistance to tacrine and almost all of the *bis*(*n*)-tacrine dimers relative to unaltered r*Pp*AChE1, with the G119S construct showing increased sensitivity to *bis*(9)-tacrine dimer compared with the unaltered r*Pp*AChE1. The F331W construct exhibited the greatest reduction in overall sensitivity to inhibition by tacrine or *bis*(*n*)-tacrine dimers.

## Discussion

This study addressed the dearth of information on the mechanism of OP and carbamate resistance in *P*. *papatasi* and mosquitoes [13, 44]. The G119S mutation of r*Pp*AChE significantly affected catalytic properties as well as enzyme sensitivity to inhibitors. The increased *K*_m_ is like the increased *K*_m_ seen in the G119S mutant of *Anopheles gambiae* AChE, reflecting impaired binding of substrate [37, 38]. High resistance ratios were also recorded for maloxon and paraoxon. Similarly to the aryl methycarbamates, these compounds acylate the active site serine (acylation site inhibitors) and extend into the oxyanion hole, where G119 is located. In contrast, significantly smaller insensitivity ratios were recorded for the pyrazol-4-yl methylcarbamates (Table 3, compounds **3**–**5**) in comparison with the other acylation site inhibitors (Table 3, compounds **1**, **2**) as previously observed with *Ag*AChE-G119S [38]. The smaller relative volume of pyrazol-4-yl core inhibitors (Fig. 1, compounds **3**–**5**) compared with aryl methylcarbamates presumably permits them to effectively enter the crowded active sites of G119S mutant *Ag*AChE and r*Pp*AChE1-G119S. The catalytic site inhibitor, tacrine, unlike carbamates and organophosphates, binds in the choline-binding site, rather than the oxyanion hole. Thus, inhibition by tacrine is relatively unaffected by the G119S mutation, and the resulting resistance ratio is only 5.8 (Table 3). Low resistance ratios were similarly observed for bivalent inhibitors (compounds **6**, **7**, and donepezil) and peripheral site inhibitors. Since neither class of inhibitor binds AChE near the G119S, the mutation does not apparently affect inhibition by these compounds.

Our results demonstrate that the single amino acid replacement orthologous to the G119S mutation producing high resistance to organophosphate and carbamate pesticides in mosquitoes also generates high level inhibition resistance in recombinant *P. papatasi* AChE1. Aryl methylcarbamates with specific targeting of arthropod pest AChEs and improved mammalian safety [33] indicate that use of the recombinant enzymes containing amino acid substitutions offers platforms for structure activity relationship (SAR) modeling and in vitro screening. The recombinant AChEs described here could be used to establish structure–activity relationships (SARs) for the rational design of potent and specific inhibitors targeting insecticide-insensitive AChEs with an enhanced mammalian safety profile [45].

Relatively more is known about how the G119S contributes to anticholinesterase resistance in mosquitoes than in sand flies. In mosquitoes, the G119S replacement generates high-level resistance to organophosphate and carbamate insecticides when homozygous, but at a high fitness cost [46–48], presumably resulting from a 30-fold reduced turnover number for substrate and 70% reduction in cholinergic activity [34]. Reduced frequency of the G119S allele over a 3 year period was reported in Lebanon, which was presumably the result of switching to pyrethroids for control of mosquitoes and the loss of the G119S allele in the absence of inhibitor selection pressure owing to fitness cost [49]. Despite its fitness cost, the ace-1 allele containing the G119S replacement is widespread throughout the world [50, 51]. The fitness cost associated with the G119S replacement may be reduced in the presence of *kdr*-resistance to pyrethroids [52] or by duplication of the ace-1 allele to permit maintenance of a heterozygous (polygenic *ace1*, homozygous G119S with paralogous F290V in duplicated *ace1*) state, essentially fixing it in the population [50–54]. Interestingly, the F290V mutation has a much lower RR than either G119S or F331W. Pesticide use in agriculture and for control of mosquitoes resulted in expansion of the *ace1* duplication in West Africa [55]. In the presence of the G119S substitution, use of pyrethroids might reduce the G119S allele frequency [49]. Alternatively, pyrethroid selection for *kdr*-based resistance might result in multiply resistant pest populations by reducing the fitness cost of the G119S allele [52]. The finding of the orthologous G119S codon polymorphism in a laboratory colony of *P. papatasi* and in wild captured African sand flies strongly suggests that a single nucleotide transversion (GGC → AGC) might readily occur, resulting in the rapid development of resistance to organophosphate insecticides in the presence of strong selection. A similar situation was reported for *Lutzomyia longipalpis* [56] involving the G119S and F331W orthologous codons, where a single nucleotide change would generate the corresponding resistance codon.

Insecticide stewardship is required to extend the useful life of organophosphate and carbamate pesticides approved for vector control by the World Health Organization (WHO) [31]. Insecticide use combining other technologies in integrated vector management might mitigate or prevent the development and fixation of the G119S replacement in susceptible sand fly and mosquito populations [57, 58]. Our study underscores the need for laboratory studies on the effects of additional mutations in PpAChE1, and evaluation of G119S orthologous codon polymorphisms in natural populations of *P. papatasi.* This approach will facilitate testing additional synthetic ligands for their efficacy against wild type and “mutant” forms of rPpAChE1, which could be augmented using artificial intelligence algorithms to improve construction and evaluation of inhibitory lead chemical structures [59–61].

## Conclusions

This study expanded knowledge of how the G119S orthologous replacement in r*Pp*AChE1 produces very high insensitivity to OP and carbamate inhibitors. This information is relevant as noted by Weill et al. because “the development of new insecticides that can specifically inhibit the G119S mutant form of acetylcholinesterase-1 will be crucial in overcoming the spread of resistance” [27]. Continued construction and expression of mutant forms facilitates the development of rapid molecular assays and additional tools to biochemically characterize the effects of mutations giving rise to organophosphate-insensitive *Pp*AChE1. The use of the recombinant and biochemically active r*Pp*AChE1 and revised molecular models described in this study could enable rapid screening in vitro and in silico to identify novel *Pp*AChE1 inhibitor ligands and allow for studies comparing the biochemical kinetics of inhibition. The addition of new molecular data on *Pp*AChE1 may also be used in artificial intelligence algorithms to refine predictive models of in vivo insecticidal activity for inhibitor discovery. The availability of r*Pp*AChE1s bioengineered with other mutations will advance mechanism-based screens to innovate selective sand fly anticholinesterases that are safer for nontarget organisms.

## Data Availability

Supporting data have been deposited in the Ag Data Commons Repository; Dataset. https://doi.org/10.15482/USDA.ADC/27893415.

## Abbreviations

AChE: Acetylcholinesterase
AcSCh: Acetylthiocholine
BuSCh: Butyrylthiocholine
DTNB: Dithiobisnitrobenzoic acid
r*Pp*AChE1: Recombinant *P. papatasi* AChE1
WT: Wild type
